# Medical students’ perception of the OSCE: development and validation of an instrument to assess formative and summative modalities

**DOI:** 10.31744/einstein_journal/2026AO1866

**Published:** 2026-05-27

**Authors:** Ariani Aparecida Rodrigues do Eiró Rosalin, Thomaz Bittencourt Couto

**Affiliations:** 1 Hospital Israelita Albert Einstein Faculdade Israelita de Ciências da Saúde Albert Einstein São Paulo SP Brazil Faculdade Israelita de Ciências da Saúde Albert Einstein, Hospital Israelita Albert Einstein, São Paulo, SP, Brazil.; 2 Hospital Israelita Albert Einstein São Paulo SP Brazil Hospital Israelita Albert Einstein, São Paulo, SP, Brazil.

**Keywords:** Problem-based learning, Exercise, Surveys and questionnaires, Health education, Simulation training, Educational measurement

## Abstract

**Objective:**

To develop, validate, and apply an instrument aimed at assessing medical students’ perceptions of the impact of an Objective Structured Clinical Examination (OSCE), both in its peer-based formative and traditional summative modalities, on clinical learning.

**Methods:**

This study used a mixed quantitative and qualitative approach. The instrument development was preceded by a literature review. Content validation was conducted by specialists through an OSCE using Pasquali's psychometric criteria (clarity, relevance, and pertinence). A pre-test with students enabled semantic and structural adjustments through triangulation with experts. For reliability, the Content Validity Coefficient and exploratory factor analysis were applied to the responses of fifth- and sixth-year students.

**Results:**

The final instrument included 22 items in three sections. All items had content validity coefficients equal to or greater than 0.80. The expert evaluations yielded coefficients of 0.924 for clarity, 0.925 for pertinence, and 0.945 for relevance. Target audience validation showed similarly high scores. Exploratory factor analysis of 96 medical students confirmed a unidimensional structure for both the formative and summative versions of the OSCE. The Kaiser-Meyer-Olkin index ranged from 0.91 to 0.93, and the Bartlett's test was significant (p<0.001). Factor loadings ranged from 0.75 to 0.98, explaining 77% of the variance. The internal consistency was excellent (Cronbach's alpha=0.97).

**Conclusion:**

This study developed a validated, reliable, and innovative instrument to assess students’ perceptions of the OSCE in health education.

## In Brief

An instrument was developed and validated to assess medical students’ perceptions of formative and summative OSCEs. The tool demonstrated strong content validity, a unidimensional structure, and excellent reliability, supporting its use in educational assessment and feedback in medical education.

## Highlights

■ Instrument developed to assess medical students’ perceptions of OSCEs.
■ Evidence of strong validity and excellent internal consistency.
■ Formative and summative OSCE modalities were also assessed.
■ Tool supports educational assessment and feedback in medical education.

## Graphical abstract

**Figure:**
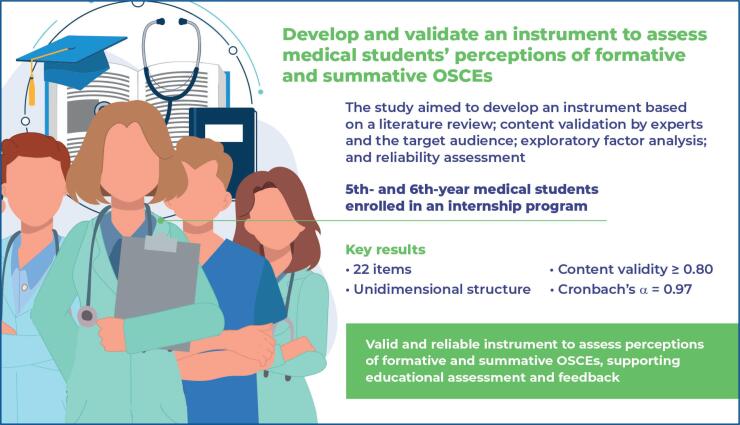

## INTRODUCTION

Active learning methodologies have become essential strategies for enhancing teaching and learning processes in medical education.^(1)^ Among these approaches, clinical simulation stands out because it provides controlled environments that realistically replicate professional scenarios and foster the development of technical, cognitive, and behavioral competencies.^(2,3)^ This method enables students to safely integrate theory and practice, promoting greater autonomy, engagement, and critical reflection.^(4)^

In the domain of practical assessment, the Objective Structured Clinical Examination (OSCE) is widely adopted by national and international institutions. Its application involves solving simulated clinical cases across standardized and sequential stations, allowing for the objective evaluation of student performance in specific competencies.^(5)^ In Brazil, this methodology has been progressively integrated into medical curricula, particularly during the clinical training phase.

Despite the growing adoption of simulations and the OSCE, the prevailing assessment model remains predominantly summative, focusing on performance measurement and grade assignment. This model often diverges from the formative and reflective principles that underpin student-centered education.^(6)^ In contrast, formative assessment has emerged as a powerful alternative that promotes structured feedback, self-reflection, and continuous improvement in clinical performance.^(7)^

Peer-assisted learning (PAL) is a collaborative strategy that complements this process by encouraging student protagonism during knowledge construction. In the context of the OSCE, the peer-based formative model has proven to be an innovative practice wherein students act as both case developers and assessors under faculty supervision. This approach supports the acquisition of clinical, communication, and evaluation skills while also fostering critical thinking, empathy, and cooperation.^(8,9)^

Despite the formative potential of this methodology, there are currently no validated instruments in Brazil that systematically assess students’ perceptions of the peer-based formative OSCE and evaluative model. The lack of specific tools limits the evaluation of the effectiveness of a strategy and hinders its pedagogical implementation. The use of reliable and validated instruments is essential for ensuring the quality of assessment processes, guiding educational interventions, and supporting research in the field.

Furthermore, existing literature lacks instruments that fully meet the criteria of an ideal outcome measurement tool for this educational context. According to Prinsen al.,^(10)^ such instruments must demonstrate content validity, internal consistency, and reliability, while also being feasible, acceptable, responsive to change, and culturally appropriate for the target population. These attributes are essential to ensure that the tool effectively captures student perceptions, supports evidence-based educational strategies, and enables comparisons across different implementation settings.

In light of this scenario, the present study aimed to develop and validate an instrument to assess medical students’ perceptions of the peer-based formative OSCE and the evaluative model implemented in clinical simulation. The creation of a robust and contextually grounded questionnaire seeks to strengthen assessment practices in medical education and promote more critical, reflective, and guideline-aligned professional training.

## OBJECTIVE

To develop, validate, and apply an instrument to assess medical students’ perception of the impact of an Objective Structured Clinical Examination, both in its peer-based formative and traditional summative modalities, on clinical learning.

## METHODS

This study followed a structured methodological approach aligned with best practices for scale development, particularly the three-phase framework proposed by Boateng et al.,^(11)^ which includes: 1) item generation, 2) content validity assessment, and 3) statistical evaluation of construct validity and reliability. The instrument was designed to assess medical students’ perceptions of the impact of peer-based formative OSCE as well as traditional summative OSCE on clinical learning. The theoretical foundation was based on the principles of formative assessment, which emphasizes active participation, self-reflection, and feedback as central components of the learning process. These principles support the development of a valid and reliable instrument, tailored to the specificities of clinical simulation environments in medical education.

### Phase 1 – Item Generation and Instrument Construction

In the first phase, item generation was conducted through an extensive integrative literature review using the MEDLINE, LILACS, and SciELO databases, with a focus on publications published between 2010 and 2023. Supplementary sources include theses, dissertations, institutional guidelines, and reference books addressing clinical simulation, peer-assisted learning, OSCE assessment, and student perceptions in health education. Although existing tools such as the National League for Nursing (NLN) Satisfaction and Self-Confidence in Learning Scale^(12)^ were reviewed, their scope did not fully address the specific competencies and contextual elements inherent to the OSCE format. Therefore, adaptations and formulation of new items are necessary because of the absence of a specific instrument in the existing literature.

Items were selected based on thematic relevance, alignment with formative assessment principles, and the potential to reflect the educational value of both summative and peer-based formative OSCE modalities. The preliminary version of the instrument was structured into three sections: Section 1: Socio-demographic profile (five items); Section 2: Perceptions of both OSCE modalities (15 Likert-scale items); and Section 3: Specific experiences related to the peer-based formative OSCE, including five Likert-type and two open-ended questions.

### Phase 2 – Content Validation and Pretesting

In the second phase, content validity was established through expert evaluation and pilot testing, in accordance with psychometric recommendations by Hernández-Nieto^(13)^ and Pasquali^(14)^ A panel of ten expert judges, all medical educators experienced in simulation-based assessment and OSCE implementation, reviewed the instrument. Using an online form, they rated each item based on clarity, relevance, and coherence, following Pasquali's criteria, and the Content Validity Coefficient (CVC) was calculated based on the method by Hernández-Nieto.^(13)^ Items with CVC <0.80 were revised according to feedback and re-evaluated.

To complement the expert analysis, the instrument underwent semantic validation via a pretest with ten fifth- and sixth-year medical students who had experienced both OSCE formats. The participants evaluated the comprehensibility and applicability of each item, contributing to linguistic and contextual adjustments. Students were excluded from the final validation cohort.

### Phase 3 – Construct validity and reliability analysis

In the third phase, the final version of the instrument was applied to a purposive sample of 120 fifth- and sixth-year medical students enrolled in a medical internship program at the study institution. The students participated in both summative and peer-based formative OSCE formats as part of their assessment matrix. The instrument was administered in person, immediately after the completion of each OSCE modality, via QR code and email access to an online form (Google Forms®). Data were collected in November 2025.

A total of 96 students responded, resulting in a response rate of 80 %. The excluded students were in external clinical rotations during the collection period.

Data analysis focused on evaluating construct validity through exploratory factor analysis (EFA) using a polychoric correlation matrix and unweighted least squares extraction, which is recommended for ordinal data with a non-normal distribution. Model adequacy was assessed using the Kaiser-Meyer-Olkin (KMO) index, Bartlett's test of sphericity, factor loadings, communalities, and Root Mean Square Residual (RMSR) index. The internal consistency was measured using Cronbach's alpha. Analyses were performed using the RStudio software (version 2023.12.0.369).

This study was approved by the Research Ethics Committee of *Hospital Israelita Albert Einstein* (CAAE: 79607624.0.0000.0071; #6.950.435) and complied with Resolution No. 466/2012 of the Brazilian National Health Council. All participants signed an informed consent form, and electronic consent was recorded in the case of student respondents.

## RESULTS

The instrument was developed and validated in three sequential stages, as described below.

### Phase 1 – Instrument construction

The construction of the questionnaire was guided by the principles of formative assessment and was based on the Student Satisfaction and Self-Confidence in Learning Scale proposed by the NLN, and adapted to the context of medical education. The formulation of the items was supported by a comprehensive review of national and international literature, with searches performed in the MEDLINE, LILACS, and SciELO databases between 2010 and 2023 as well as consultations of theses, dissertations, conference proceedings, and relevant academic books.

The extracted material was grouped by thematic similarity, allowing for the categorization of content and the development of items that reflected the main dimensions relevant to assessing the impact of the OSCE on clinical learning. The initial version of the instrument comprised 21 items organized into three sections: 1) Five sociodemographic questions (age, gender, academic year, state of origin, and intended medical specialty); 2) 15 closed-ended items using a five-point Likert scale, assessing general perceptions of both summative and formative OSCE modalities; 3) One open-ended question about the formative OSCE experience.

### Phase 2 – Content validation with expert judges and Validation with target audience

The instrument was evaluated by a panel of 10 expert judges, all medical educators with extensive experience in clinical simulation and OSCE assessment (Table 1).

**Table 1:** Profile of expert judges who participated in content validation of the instrument (n=10)

Characteristic		Result
Age (years)		
	Mean	42.8±9.4
	Range	33-61
Sex - %		
	Male	60
	Female	40
Academic qualification - %		
	Doctoral degree	40
	Master's degree	40
	Lato sensu specialization	20
Teaching experience (years)		
	Mean	13.2
Experience with active methodologies - %		80
Experience with clinical simulation - %		90

Item analysis followed the psychometric criteria proposed by Pasquali (clarity, relevance, and pertinence), and the CVC was calculated using Hernández-Nieto's formula. All 21 evaluated items obtained CVC ≥0.80 in all dimensions, with overall averages of clarity (0.924), pertinence (0.925), and relevance (0.945).

During the qualitative review, the judges recommended that the original open-ended question be divided into two specific questions: one addressing the perceived learning experience during the peer-based formative OSCE and another focused on teamwork during the simulation. This change resulted in the final version of 22 items. Additional recommendations have been incorporated through minor wording and structural revisions. Detailed results of this stage are listed in table 1.

### Pilot Testing with the Target Audience – Instrument Refinement Phase

In the third stage, the revised questionnaire was administered to six medical students in their 5th and 6th academic years, all of whom had previously participated in both OSCE formats. Four participants were female, with a mean age of 27.5 years. The purpose of this phase was to assess the instrument's semantic clarity, practical applicability, and linguistic adequacy for the intended audience.

The content validity analysis also showed high CVC scores, with averages of 0.977 for clarity, 0.980 for pertinence, and 0.986 for relevance. All items presented CVC ≥0.80 across the evaluated dimensions. Feedback from participants led to minor final adjustments, resulting in a final validated version of the instrument. The outcomes of this stage are summarized in table 2.

**Table 2:** Content validity coefficient of the items evaluated by expert judges

Item	CVC Clarity	CVC Pertinence	CVC Relevance	Interpretation		
				Clarity	Pertinence	Relevance
1	09	0.9	0.925	Acceptable	Acceptable	Acceptable
2	0.95	0.925	0.95	Acceptable	Acceptable	Acceptable
3	0.95	0.925	0.975	Acceptable	Acceptable	Acceptable
4	0.875	0.9	0.9	Acceptable	Acceptable	Acceptable
5	0.975	0.825	1	Acceptable	Acceptable	Acceptable
6	0.875	0.975	0.875	Acceptable	Acceptable	Acceptable
7	0.925	0.9	0.95	Acceptable	Acceptable	Acceptable
8	0.9	0.925	0.95	Acceptable	Acceptable	Acceptable
9	0.975	0.975	0.975	Acceptable	Acceptable	Acceptable
10	0.95	0.925	0.975	Acceptable	Acceptable	Acceptable
11	0.925	0.975	0.95	Acceptable	Acceptable	Acceptable
12	0.95	1	0.975	Acceptable	Acceptable	Acceptable
13	0.925	0.925	0.95	Acceptable	Acceptable	Acceptable
14	0.875	0.85	0.925	Acceptable	Acceptable	Acceptable
15	0.85	0.875	0.9	Acceptable	Acceptable	Acceptable
16	0.875	0.9	0.9	Acceptable	Acceptable	Acceptable
17	0.925	0.925	0.925	Acceptable	Acceptable	Acceptable
18	0.925	0.925	0.95	Acceptable	Acceptable	Acceptable
19	0.975	0.95	0.95	Acceptable	Acceptable	Acceptable
20	0.875	0.925	0.95	Acceptable	Acceptable	Acceptable
21	1	1	1	Acceptable	Acceptable	Acceptable
CVC total	0.924	0.925	0.945			

CVC: Content Validity Coefficient.

### Phase 3 – Validation with the Target Audience – Exploratory Factor Analysis

The internal structure of the instrument was evaluated through exploratory factor analysis using a polychoric correlation matrix and unweighted least squares extraction method, recommended for ordinal variables and non-normal data distributions.^(13)^ Data suitability for factor analysis was confirmed by excellent Kaiser-Meyer-Olkin indices (KMO=0.91 for the formative OSCE and 0.93 for the summative) and a significant Bartlett's test of sphericity (p<0.01 for both).^(14)^

Parallel analysis indicated the extraction of a single factor for both scales (formative and summative), thereby supporting the unidimensionality of the instrument.^(15)^ The factor loadings ranged from 0.75 to 0.98, with communality above 0.60. A single factor explained 77% of the total variance in both versions. Model fit was satisfactory, with a Root Mean Square Residual (RMSR) of 0.05, within the desirable range (<0.08).^(16)^ These findings are detailed in table 3, which presents the factor loadings and communality for each item. Factor loadings were rotated using the oblimin method to simplify the interpretation.

**Table 3:** Content validity coefficient of the items evaluated by the target audience

Item	CVC Clarity	CVC Pertinence	CVC Relevance	Interpretation		
				Clarity	Pertinence	Relevance
1	1.000	1.000	1.000	Acceptable	Acceptable	Acceptable
2	1.000	1.000	1.000	Acceptable	Acceptable	Acceptable
3	1.000	1.000	1.000	Acceptable	Acceptable	Acceptable
4	0.958	1.000	1.000	Acceptable	Acceptable	Acceptable
5	0.958	0.833	0.958	Acceptable	Acceptable	Acceptable
6	0.958	1.000	0.917	Acceptable	Acceptable	Acceptable
7	1.000	1.000	1.000	Acceptable	Acceptable	Acceptable
8	0.958	0.958	0.958	Acceptable	Acceptable	Acceptable
9	1.000	1.000	1.000	Acceptable	Acceptable	Acceptable
10	1.000	1.000	1.000	Acceptable	Acceptable	Acceptable
11	1.000	1.000	1.000	Acceptable	Acceptable	Acceptable
12	1.000	1.000	1.000	Acceptable	Acceptable	Acceptable
13	0.958	0.958	0.958	Acceptable	Acceptable	Acceptable
14	0.958	0.917	0.958	Acceptable	Acceptable	Acceptable
15	0.917	0.958	0.958	Acceptable	Acceptable	Acceptable
16	0.958	1.000	1.000	Acceptable	Acceptable	Acceptable
17	0.958	0.958	1.000	Acceptable	Acceptable	Acceptable
18	1.000	1.000	1.000	Acceptable	Acceptable	Acceptable
19	1.000	1.000	1.000	Acceptable	Acceptable	Acceptable
20	0.958	1.000	1.000	Acceptable	Acceptable	Acceptable
21	1.000	1.000	1.000	Acceptable	Acceptable	Acceptable
CVC total	0.977	0.980	0.986			

CVC: Content Validity Coefficient.

The polychoric correlation matrix showed strong interitem correlations, further supporting factorability and scale coherence. A complete summary of these correlations is presented in table 4.

**Table 4:** Factor loadings and communalities considering a single factor

Item	Summative OSCE		Formative OSCE	
	Factor loadings*	Communalities	Factor loadings*	Communalities
Q1	0.88	0.77	0.79	0.63
Q2	0.82	0.67	0.82	0.67
Q3	0.81	0.66	0.78	0.61
Q4	0.75	0.56	0.82	0.67
Q5	0.87	0.75	0.82	0.68
Q6	0.89	0.78	0.85	0.72
Q7	0.94	0.89	0.92	0.84
Q8	0.91	0.83	0.92	0.85
Q9	0.82	0.68	0.77	0.60
Q10	0.80	0.64	0.86	0.73
Q11	0.94	0.88	0.96	0.92
Q12	0.95	0.90	0.98	0.96
Q13	0.96	0.91	0.98	0.95
Q14	0.89	0.80	0.93	0.87
Q15	0.91	0.83	0.95	0.89

* The factor loadings were rotated using the oblimin method to simplify the interpretation.

The instrument's internal consistency was considered excellent; Cronbach's alpha for both versions was 0.97, indicating a strong inter-item correlation and psychometric robustness.^(17)^ No item negatively affected the scale as all items presented item-total correlations between 0.71 and 0.94. These results are presented in table 5, which details the internal consistency coefficients for each item.

**Table 5:** Internal consistency. Polychoric inter-item correlation matrix

Item	Summative OSCE		Formative OSCE	
	α if item deleted	Correlation with total score	α if item deleted	Correlation with total score
Q1	0.96	0.83	0.97	0.78
Q2	0.97	0.78	0.97	0.78
Q3	0.97	0.77	0.97	0.71
Q4	0.97	0.71	0.97	0.75
Q5	0.96	0.82	0.97	0.78
Q6	0.96	0.82	0.97	0.74
Q7	0.96	0.90	0.96	0.88
Q8	0.96	0.89	0.96	0.88
Q9	0.97	0.77	0.97	0.72
Q10	0.97	0.76	0.96	0.80
Q11	0.96	0.90	0.96	0.91
Q12	0.96	0.90	0.96	0.93
Q13	0.96	0.90	0.96	0.94
Q14	0.96	0.85	0.96	0.89
Q15	0.96	0.87	0.96	0.89

The inter-item correlations demonstrated strong and consistent associations among the scale items, supporting the instrument's internal consistency as shown in table 6.

**Table 6:** Internal consistency coefficients for each item. Polychoric correlation matrix

Item	Q1	Q2	Q3	Q4	Q5	Q6	Q7	Q8	Q9	Q10	Q11	Q12	Q13	Q14
Summative OSCE														
Q2	0.79													
Q3	0.70	0.78												
Q4	0.67	0.65	0.64											
Q5	0.76	0.75	0.67	0.68										
Q6	0.82	0.73	0.74	0.70	0.77									
Q7	0.84	0.75	0.72	0.68	0.83	0.88								
Q8	0.76	0.73	0.77	0.81	0.79	0.79	0.92							
Q9	0.72	0.72	0.67	0.71	0.71	0.70	0.81	0.81						
Q10	0.81	0.62	0.71	0.64	0.65	0.65	0.65	0.70	0.59					
Q11	0.82	0.73	0.70	0.63	0.84	0.85	0.89	0.80	0.74	0.77				
Q12	0.83	0.74	0.75	0.63	0.84	0.80	0.91	0.86	0.79	0.75	0.94			
Q13	0.77	0.75	0.76	0.67	0.85	0.78	0.91	0.86	0.81	0.77	0.94	0.94		
Q14	0.75	0.70	0.72	0.62	0.75	0.82	0.83	0.79	0.65	0.79	0.89	0.82	0.90	
Q15	0.76	0.68	0.74	0.59	0.76	0.83	0.84	0.78	0.71	0.79	0.92	0.92	0.92	0.91
**Formative OSCE**														
Q2	0.66													
Q3	0.57	0.67												
Q4	0.58	0.72	0.72											
Q5	0.75	0.69	0.70	0.62										
Q6	0.69	0.75	0.53	0.73	0.72									
Q7	0.77	0.69	0.74	0.76	0.83	0.78								
Q8	0.74	0.78	0.79	0.82	0.73	0.66	0.88							
Q9	0.60	0.69	0.80	0.77	0.59	0.52	0.66	0.82						
Q10	0.60	0.64	0.71	0.70	0.68	0.68	0.74	0.85	0.75					
Q11	0.72	0.74	0.70	0.75	0.81	0.90	0.90	0.85	0.70	0.85				
Q12	0.80	0.80	0.70	0.77	0.77	0.87	0.91	0.89	0.70	0.82	0.96			
Q13	0.81	0.81	0.71	0.77	0.79	0.86	0.87	0.87	0.70	0.81	0.95	0.98		
Q14	0.70	0.75	0.71	0.71	0.72	0.85	0.79	0.80	0.67	0.88	0.94	0.94	0.96	
Q15	0.79	0.73	0.64	0.72	0.76	0.83	0.89	0.85	0.63	0.81	0.93	0.97	0.97	0.94

The final version of the instrument comprised 22 items structured into three thematic sections designed to comprehensively assess medical students’ perceptions of formative and summative OSCE modalities. Section 1 addresses the general aspects of the OSCE experience and includes 15 closed-ended items evaluated on a five-point Likert scale, focusing on the competencies developed, clarity of instructions, realism of scenarios, adequacy of resources, feedback quality, and the overall impact on clinical learning. Section 2 explores students’ specific perceptions regarding the peer-based formative OSCE, with five closed-ended items covering knowledge construction, assessment capacity, debriefing, confidence, and perspective-taking. Finally, Section 3 presents two open-ended questions that allow participants to express their reflections on the learning process and teamwork experienced during formative OSCE. Thus, the instrument integrates both quantitative and qualitative elements, aiming to capture the complexity and depth of educational experiences in simulated clinical assessments.

Instrument – Medical Students’ Perceptions of OSCE (Formative and Summative).

Instruction: Please mark the number that best represents your level of satisfaction with each statement. (Scale: 1=Very Unsatisfied, 2=Partially Unsatisfied, 3=Neither Satisfied nor Unsatisfied, 4=Partially Satisfied, 5=Very Satisfied).

Section 1 – General Perception of OSCE Modalities (Summative and Formative – Table 1S, Supplementary Material).

## DISCUSSION

Based on the pilot test and the initial administration of the questionnaire, the CVC scores obtained during the development and validation phases demonstrated strong consistency among the evaluators, including both experts and students. These findings support the robustness of the instrument developed to accurately capture medical students’ perceptions of the OSCE in its formative and summative modalities, thus reinforcing its potential use in similar educational settings.

The high CVC values obtained from expert evaluation – clarity (0.924), pertinence (0.925), and relevance (0.945) – indicated that the items were appropriate in terms of language, evaluative focus, and alignment with the research objectives. These results are consistent with the literature, which establishes 0.80 a minimum threshold for acceptable content validity.^(12)^ Furthermore, the qualitative suggestions from the expert panel, particularly regarding terminology and item objectivity, underscored the importance of expert involvement in the development of valid instruments, as highlighted in studies on psychometric assessment in health education.^(14,18,19)^

Validation with the target audience also yielded excellent CVC scores for clarity (0.977), pertinence (0.980), and relevance (0.986), indicating that the students found the items clear, appropriate to their context, and reflective of their experiences with the OSCE. This positive feedback reinforces not only the instrument's semantic validity but also its cultural and pedagogical appropriateness.

The unidimensional structure identified through exploratory factor analysis, both for the summative and formative OSCE versions, indicated that the 15 items of the instrument consistently measured a single construct: student satisfaction with the assessment experience. This finding is reinforced by the high internal consistency (α=0.97) and the statistical adequacy demonstrated by the KMO index (0.93) and Bartlett's test (p<0.05). These results align with those of recent studies that have used similar psychometric parameters to validate scales in medical education settings and reported strong cohesion among the evaluated items.^(20,21)^

The application of parallel analysis, which confirmed the instrument's unidimensionality, further supported the scale's precision in capturing students’ perceptions of formative experiences within the OSCE. Considering that the instrument was tested in an innovative setting, where students not only participated in the exam but also engaged in scenario development, peer assessment, and feedback sessions guided by a faculty expert, it can be inferred that such active involvement contributed to the homogeneity of responses. This is because students tend to perceive experience as a single integrated process, leading to the concentration of evaluations under one factor.^(22)^

Additionally, the predominance of responses at the upper end of the scale suggests a high level of satisfaction but also indicates the need for continuous monitoring of potential ceiling effects, especially in different institutional contexts. Nevertheless, the instrument has proven to be both robust and sensitive, with a structure that aligns with the findings of Zhuang et al., who validated a similar scale for medical students in China and Guilarte et al., who assessed academic satisfaction in professional training contexts. These studies confirm that, when well structured, assessment instruments can effectively capture students’ experiences, even in educational environments characterized by high variability.^(23,24)^

In this regard, the validated questionnaire can contribute significantly to future research on assessment strategies in medical education and health-related programs, enabling longitudinal comparisons and inter-institutional applications. The use of instruments with psychometric validity strengthens the scientific foundation of educational assessment, which is an essential component for fostering quality and innovation in medical curricula.^(23)^

Moreover, the results from the questionnaire application revealed high acceptance of the peer-based formative OSCE by students, who reported greater satisfaction with this experience than with the traditional summative model. This positive perception highlights the effectiveness of formative OSCE as a pedagogical tool to consolidate clinical learning, stimulate critical thinking, and enhance students’ self-confidence, which are key competencies in the medical training process.^(24,25)^

The slightly higher factor loadings and Cronbach's alpha values observed for the items related to the peer-based formative OSCE may be explained by the learning environment inherent to this modality. Unlike summative OSCE, which is usually associated with higher levels of anxiety and performance pressure, formative OSCE emphasizes feedback and skill development. In this context, students tend to engage in more reflective and consistent responses, which increases the homogeneity of their answers, thereby strengthening internal consistency and factor correlations. Greater satisfaction with peer-based formative OSCE may also indicate higher engagement and motivation, which have been associated with improved knowledge retention and long-term learning outcomes. Pedagogically, these findings reinforce the value of incorporating peer-assisted formative practices into curricula as they foster student protagonism, feedback literacy, and collaborative learning, which are considered essential in contemporary medical training.

It is important to note, however, that the NLN Student Satisfaction and Self-Confidence in Learning Scale was not originally designed to evaluate OSCE experience. Although it has been widely used and validated in different contexts, including the Portuguese version,^(26)^ adaptations are required to adequately capture the specific dimensions of students’ perceptions in structured clinical examinations. This limitation highlights the relevance of developing and validating instruments specifically tailored to OSCE assessments in medical education.

## CONCLUSION

Medical students’ perception of clinical assessment strategies, such as the OSCE, is a relevant indicator for improving teaching quality in medical education. In this study, a specific instrument was developed and validated to measure students’ experiences with both peer-based formative and traditional summative modalities of the OSCE. The results demonstrated strong psychometric properties with high content validity indices reported by both experts and the target audience.

The questionnaire proved appropriate in terms of clarity, pertinence, and relevance of the items, establishing it as a reliable tool for assessing the subjective dimensions of clinical learning. Furthermore, its application revealed significant acceptance of peer-based formative OSCE among students, highlighting its pedagogical potential as an active teaching strategy.

These findings reinforce the importance of incorporating innovative assessment methodologies into medical education, particularly those that promote student engagement, qualified feedback, and learner autonomy throughout the educational process.

## Data Availability

The underlying content is contained within the manuscript.
