# Supplementary material for: Medical students’ perception of the OSCE: development and validation of an instrument to assess formative and summative modalities
Source: Einstein (Sao Paulo). 2026 May 27;24:eAO1866. doi: 10.31744/einstein_journal/2026AO1866 (PMC13399294; doi:10.31744/einstein_journal/2026AO1866)
Supplement: SUPPLEMENTARY MATERIAL [file 2317-6385-eins-24-eAO1866-Suppl1.pdf]

SUPPLEMENTARY MATERIAL

Medical students' perception of the OSCE: development and validation of an instrument to assess formative and summative modalities

Ariani Aparecida Rodrigues do Eiró Rosalin, Thomaz Bittencourt Couto

DOI: 10.31744/einstein_journal/2026AO1866

Table 1S. Description of the items in the instrument for assessing medical students' perceptions of formative and summative OSCE modalities

Item	Statement	1	2	3	4	5
1	The activities carried out during the OSCE contributed to the development of competencies (knowledge, skills, attitudes, and values).	☐	☐	☐	☐	☐
2	The instructions provided prior to the activities were clear and sufficient for my preparation.	☐	☐	☐	☐	☐
3	The time available at each station was adequate to safely and efficiently complete the proposed tasks.	☐	☐	☐	☐	☐
4	The human (facilitator, assessor, and assistant professor) and material resources were appropriate for conducting the activities.	☐	☐	☐	☐	☐
5	The scenarios presented during the exam realistically reflected the clinical situations encountered in the internship field.	☐	☐	☐	☐	☐
6	The proposed exam enabled the practice and improvement of key clinical skills necessary for my training.	☐	☐	☐	☐	☐
7	The scenarios presented helped me to develop clinical reasoning and decision-making in different situations.	☐	☐	☐	☐	☐
8	The learning objectives set for these activities were clear and achieved by the end of the exam.	☐	☐	☐	☐	☐
9	The feedback received during and after the activities was appropriate and contributed to my learning.	☐	☐	☐	☐	☐
10	The environment provided by the team was welcoming and helped me perform the activities calmly.	☐	☐	☐	☐	☐
11	The experience gained during the exam increased my confidence in handling real clinical situations.	☐	☐	☐	☐	☐
12	I believe this experience was important for my development as a future healthcare professional.	☐	☐	☐	☐	☐
13	The assessments helped improve my ability to apply theoretical knowledge to practical situations.	☐	☐	☐	☐	☐
14	I feel I was able to adequately demonstrate my clinical skills and critical thinking during the proposed activities.	☐	☐	☐	☐	☐
15	The OSCE, as an active learning strategy, helped consolidate knowledge that I would hardly have acquired through traditional teaching methods alone.	☐	☐	☐	☐	☐

Section 2 – Specific Perception of the Peer-Based Formative OSCE

Item	Statement	1	2	3	4	5
1	Developing the clinical case for the formative OSCE helped deepen my knowledge of the subject and enhance my clinical reasoning skills.	☐	☐	☐	☐	☐
2	As an assessor of my peers during the formative OSCE, I was able to clearly identify and evaluate essential competencies.	☐	☐	☐	☐	☐
3	The debriefing and discussion process guided by the student assessors and expert facilitator expanded my understanding of protocols and guidelines.	☐	☐	☐	☐	☐
4	Participating in the formative OSCE helped boost my confidence in applying theoretical knowledge to real-life practical scenarios.	☐	☐	☐	☐	☐
5	The role-switching between being a student assessor and participant in the formative OSCE was relevant in developing different learning perspectives.	☐	☐	☐	☐	☐

Section 3 – Open-Ended Questions

As a participant in the peer-based formative OSCE, what is your perception regarding the development of your learning process?

Response: ______________________________________

How would you describe your experience in the formative OSCE regarding teamwork?

Response: ______________________________________

Source: Adaptade from:

Source: Developed by the authors.
